# Lifting the Veil of Darkness: Thermal Technology Facilitates Collection of Flight‐Initiation Distances by Night

**DOI:** 10.1002/ece3.70450

**Published:** 2024-11-19

**Authors:** Anthony R. Rendall, Roan D. Plotz, Kaori Yokochi, Joel Krauss, Aaron Pengelly, Sam A. Di Stefano, Sarah Swindell, Kithsiri Ranawana, Dulan R. Vidanapathirana, Michael A. Weston

**Affiliations:** ^1^ School of Life and Environmental Sciences, Faculty of Science, Engineering and the Built Environment Deakin University Geelong Victoria Australia; ^2^ Applied Ecology and Environmental Change Research Group, Institute for Sustainable Industries and Liveable Cities Victoria University Footscray Park Victoria Australia; ^3^ Department of Zoology, Faculty of Science University of Peradeniya Peradeniya Sri Lanka; ^4^ Herpetological Foundation of Sri Lanka Wattala Sri Lanka; ^5^ Deakin Marine Research and Innovation Centre, School of Life and Environmental Science Deakin University Geelong Victoria Australia

**Keywords:** animal behaviour, escape, human‐wildlife interactions, methodological application, nocturnal

## Abstract

Flight‐Initiation Distance (FID)—a direct measure of an individual animal's escape response—is a widely used method to study escape ecology in fauna. The technique has primarily been applied to bird species that are active by day. Indexing the escape behaviour of nocturnal species has been limited due to the need for light to detect and observe animals which confounds behavioural responses. We demonstrate the use of existing high‐end thermal technology to facilitate standardised, un‐biased, nocturnal FIDs in small and large, terrestrial and arboreal animals, which feature initial separation (starting) distances which are the same by day and night. We provide the following (1) method for collecting FIDs by night which specifically addresses solutions to novel challenges associated with collecting these by night, (2) report of the FIDs of some strictly nocturnal bird and mammal species and compare diurnal and nocturnal FIDs for some species, (3) demonstration that the positive daytime relationship between FID and Starting Distance also occurs by night, and (4) minimum sample size threshold for quantifying escape responses and how these vary when sampling the FIDs of different animal species by night. We demonstrate the capacity to conduct nocturnal FIDs on a broad range of taxa not previously studied. We recommend 25–50 samples are needed to accurately quantify a species escape response in a particular context. Our method expands the capacity to understand how species escape by night, a critical period during which many predator–prey interactions occur.

## Graphical abstract

Flight‐Initiation Distance (FID) measures an animal's escape response and is widely used to study escape ecology on diurnal animals. It is difficult to detect and observe nocturnal animals due to the need for light. Using existing high‐end thermal technology, we demonstrate a novel method for obtaining nocturnal FIDs for terrestrial and arboreal animals. We show that a wide range of taxa can be studied with nocturnal FIDs and species escape response in a particular context requires a minimum of 25–50 samples. By using our method, we provide an opportunity to better understand how predators and prey interact at night.

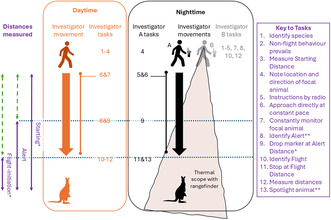

## 1 Introduction

There is a critical need to broaden nighttime ecology to address fundamental questions about the differences between daytime and nighttime ecology (Gaston 2019). The nighttime environment is under increasing anthropogenic pressure including through altered predator–prey interactions; expanding our knowledge is therefore critical for effective conservation (Filla et al. 2017; Gaston 2019). Escape is an important aspect of life history, and the burgeoning field of escape ecology has revolutionised our understanding of the evolution of and plasticity of predator avoidance and assisted conservation biologists to manage causal threatening processes such as human disturbance (Cooper and Blumstein 2015). Flight‐Initiation Distance (FID), evoked by human approaches to focal animals, is a standardised, direct and relative measure of an individual's propensity to escape and can therefore be used to assess how this may change in response to, for example, anthropogenic pressures (Fernández‐Juricic et al. 2005; Mikula et al. 2023; Thibault et al. 2020), landscape contexts (Hall et al. 2020; Radvan, Rendall, and Weston 2023), social organisation (Blumstein, Evans, and Daniel 1999; Shuai et al. 2024), with biogeography (Cooper, Pyron, and Garland 2014; Weston et al. 2021), or even with historical human disturbances (Gnanapragasam et al. 2021). FIDs have been conducted on a diverse range of fauna, most commonly on birds (Guay et al. 2016; Weston et al. 2012), but also reptiles (Samia et al. 2016), mammals (Ortiz‐Jimenez et al. 2022) and invertebrates (Harbour et al. 2019). Almost no FIDs are available by night, because of prevailing key methodological limitations, which have restricted sampling to periods during which human vision is sufficient to observe wildlife and conduct other aspects of sampling.

Collection of FIDs relies on a series of human visual capacities which are limited by darkness. An observer must detect and identify an animal at a distance beyond which the animal responds; distances must be measured; and a standardised walking approach over often uneven terrain must be made directly to the focal animal which is constantly and simultaneously monitored for response (Blumstein 2003). The use of visual aids such as spotlights (white, red, or green, e.g., West et al. 2017) or night vision (e.g., Ross, Lawes, and Letnic 2022) represents un‐natural, evolutionarily novel, stimuli which might evoke atypical predator escape responses, such as freezing until very close proximity (Wolf and Croft 2012) or fleeing earlier due to heightened vigilance in response to light. Night vision goggles contain either infrared or near‐infrared light sources (wavelengths > 700 nm) which can be seen by some invertebrate, reptile and mammal species (e.g., Newbold and King 2008; Goris 2011) and thus may confound nocturnal FIDs in the same ways spotlights would. Thus, nocturnal FIDs are virtually unavailable, and those that are available have been compelled to use spotlights and customised techniques, which likely influence all aspects of the investigator approach and animal response, including detection of the focal animal at a sufficient distance (e.g., West et al. 2017; Ross, Lawes, and Letnic 2022; Aikawa and Saito 2023). Although we note studies of collision avoidance behaviour of wildlife understandably and intentionally use spot or headlights (e.g., DeVault, Seamans, and Blackwell 2020).

Nocturnal escape behaviour is likely a critical determinant of survival of predators and prey. While chronically understudied by night, in many cases antipredator and predator behaviour likely change by day and night (Filla et al. 2017; Gaston 2019). Day and night represent different challenges to predator and prey species and may differ with respect to activity, behaviour and habitat/refuge selection, effectiveness of different sensory modalities used to hunt (predators) and detect and identify predators (prey), as well as the relevance of social cues for escape, effectiveness of crypsis and hiding and possibly even energy states (Filla et al. 2017; Perea‐Rodríguez et al. 2022; Richter et al. 2020), by day and night, among other differences. Nocturnality itself is an adaptation of both predators and prey, and nocturnal interactions between predators and prey may drive evolutionary arms races more than those by day. Thus, the lack of understanding of nocturnal antipredator behaviour inhibits the understanding of escape and the assumption that diurnal escape behaviour reflects nocturnal escape behaviour is a foundational yet untested assumption in much escape ecology.

Recent advances in thermal technology permit effective nocturnal ecological research (Gaston 2019). Thermal technology senses and visualises differences in heat and does not emit light in visible or infrared ranges that could be detected by wildlife. With advances in quality and cost‐effectiveness, the capacity to conduct unbiased, standardised FIDs by night exists. Here, we outline a method and technology required to perform FIDs safely and effectively by night. Through trial and learning, we developed and presented a practical protocol for conducting nocturnal multispecies FIDs which holds the promise of expanding the realm of fear ecology research (*sensu* Gaston 2019).

## 2 Methods

**FIGURE 1:**
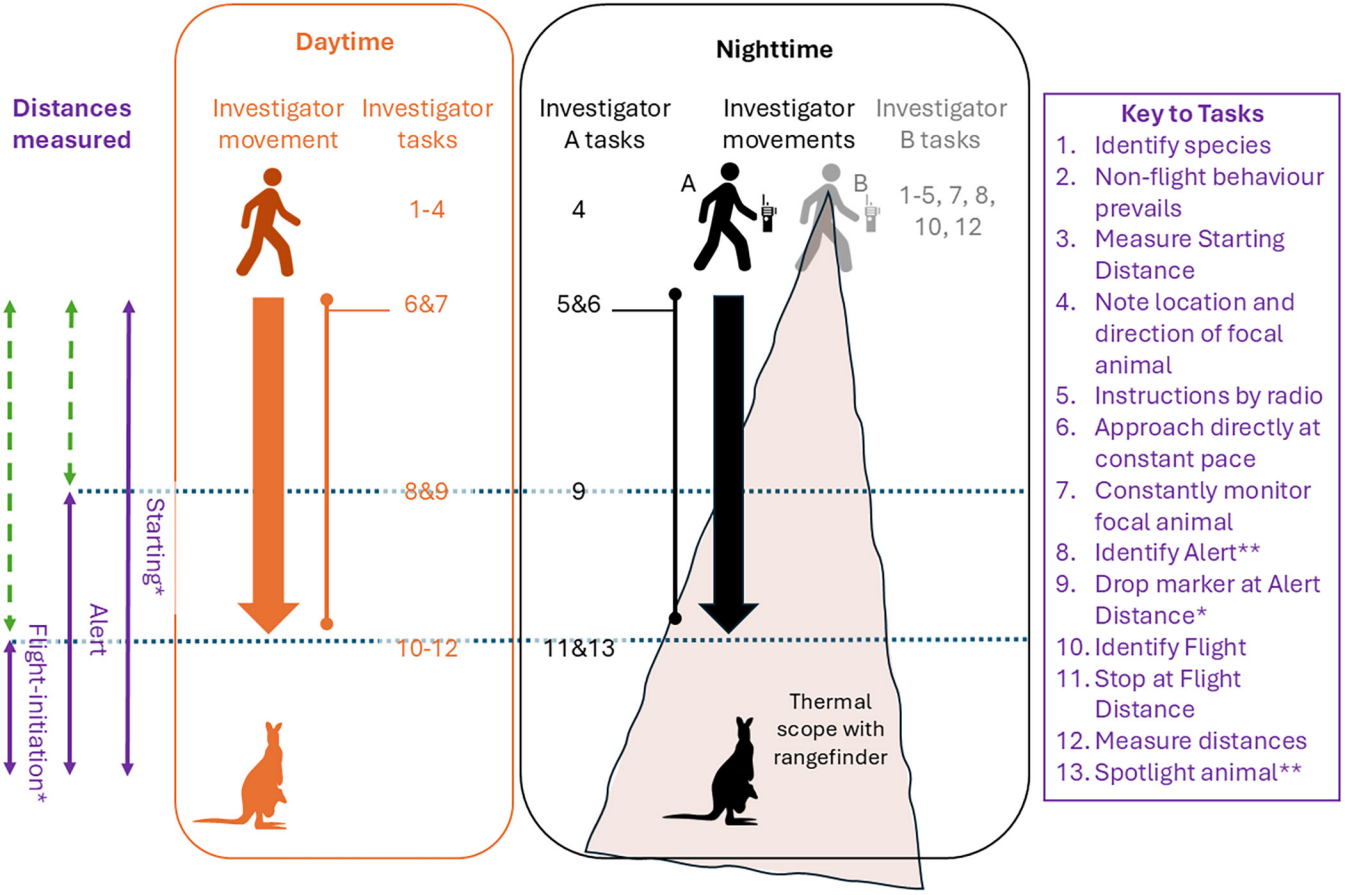
Schematic comparison of protocols to collect flight‐initiation distances by day (middle) and night (right), relative to distances measured and phase of procedure. “Complementary” distances (green dashed arrows) are those which can be readily measured and used to calculate required distances (purple). *Indicates distances required and **indicates procedures which are performed only if relevant/required.

Nocturnal FIDs involve the same fundamental processes as when conducted diurnally (diurnal methods are well described, e.g., Weston et al. 2012 and summarised in Figure 1). Briefly, an investigator identifies a focal animal exhibiting undisturbed behaviours, records Starting Distance (StD; the initial distance to the focal animal) and then walks at a constant pace (~1 ms^−1^) directly towards the focal animal recording any distinct Alert Distance (if any) and recording FID (initiation of any movement associated with commencing escape), although we note that collecting nocturnal FIDs (and some diurnal FIDs with long StDs) ideally involves two investigators—an ‘approacher’ (A) and an ‘observer’ (B) (Figure 1)—who divide the abovementioned tasks and communicate quietly via two‐way radios with earpieces (e.g., GME 5‐W UHF radios with headsets). The narrow field of view of night vision equipment (which can also zoom in to distant animals) means walking at constant pace (over uneven terrain) while monitoring animal response is impractical in our experience. The observer therefore directs the approacher to leave markers or to stop when AD and FID are detected and records all distances using the in‐built laser‐range finder (videos available at: https://doi.org/10.5281/zenodo.11081716), often by measuring complementary distances (Figure 1, Video 1). All observers aim to walk at a steady pace, although slight variations could occur with different landscape contexts or individuals. Lethlean et al. (2017) showed FID and response modality varied with joggers compared to walkers; however, we suggest our variation in speed is negligible in comparison. In thick habitats, when rangefinders return unreliable distances (e.g., returns off vegetation), the approacher may need to pace some distances, at the direction of the observer, at the conclusion of the approach. The approach occurs with minimal sound and no light cues, a single person approaching at constant pace, and is readily standardisable (Cooper and Blumstein 2015); we consider these comparable to diurnal approaches (see Section 3).

**VIDEO 1:** Human approach towards a group of Spotted Deer (*Axis axis*) demonstrating a distinct flight‐initiation distance.

**FIGURE 2:**
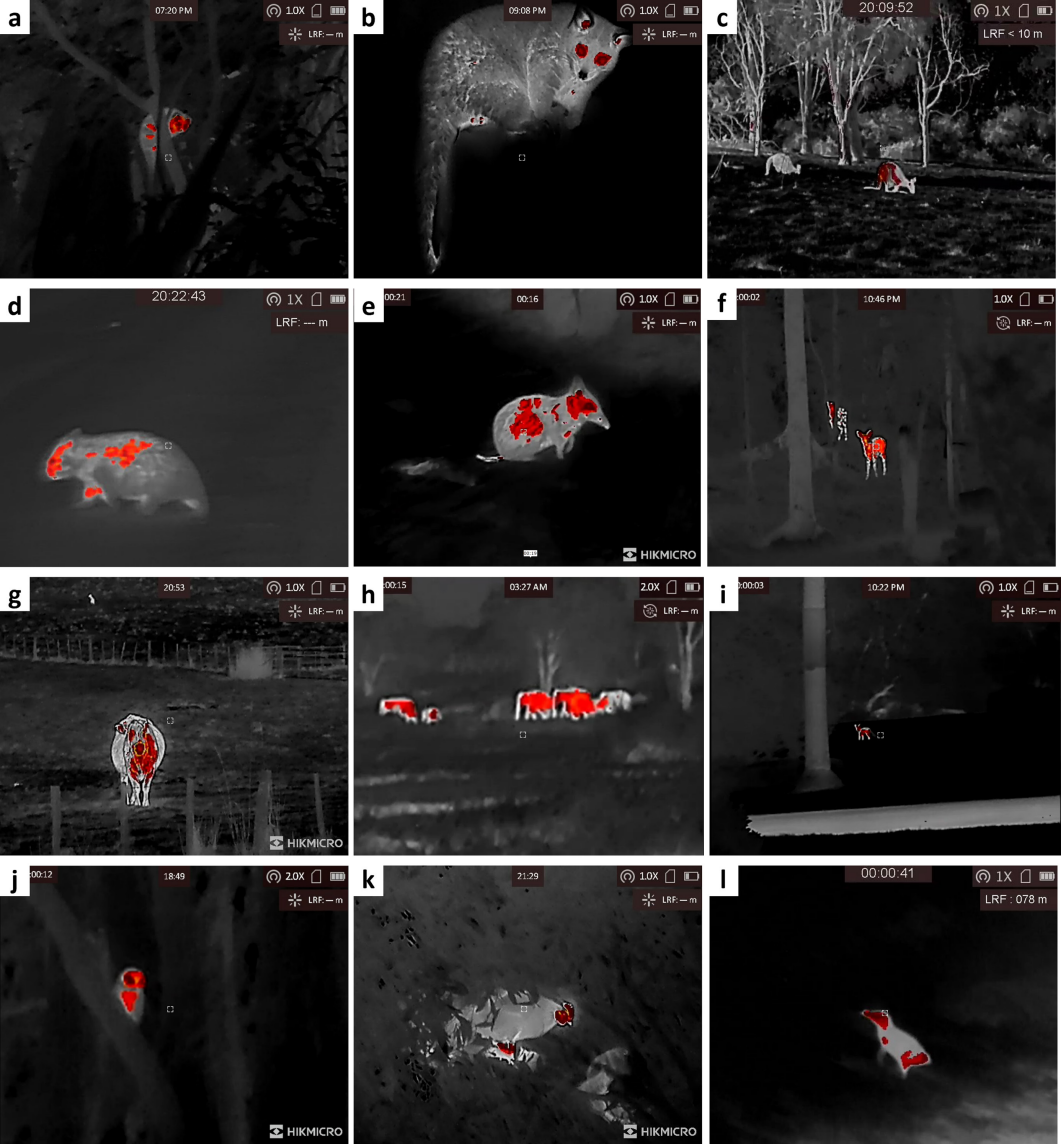
Example images of mammalian and avian species viewed through a thermal monocular. (a) Common Ringtail Possum, *Pseudocheirus peregrinus*, (b) Common Brushtail Possum, *Trichosurus vulpecula*, (c) Eastern Grey Kangaroo, *Macropus giganteus*, (d) Common Wombat, *Vombatus ursinus*, (e) Eastern Barred Bandicoot, *Perameles gunnii*, (f) Spotted deer, *Axis axis*, (g) Cattle, *Bos taurus*, (h) Asian Elephant, *Elephas maximus*, (i) Fishing Cat, *Prionailurus viverrinus*, (j) Tasmanian Morepork, *Ninox novaeseelandiae*, (k) Chicken, *Gallus gallus domesticus* and (l) Little Penguin, *Eudyptula minor*.

To aid in species identification, observation and distance measurement we use high‐quality thermal equipment (HIKMICRO Gryphon GH25L thermal monocular; Hangzhou Microimage Software Co. Ltd., China). These devices provided clear images (Figure 2), enabled recordings to be taken when identification was ambiguous and simultaneously permitted measurement of distances, while enabling the animals' movements and behaviours to be tracked. The quality of this equipment meant key characteristics and movements were easily visible meaning a vast majority of species could then be identified based on gait (see videos at https://doi.org/10.5281/zenodo.11081835), foraging behaviour and body characteristics (Figure 2). If identification remained ambiguous (i.e., rodents), we spotlighted the animal after their escape to confirm identification. Where identification was not able to be confirmed, the datum was discarded. Spotlighting by the approacher after escape commences can also provide details of sex and age of the focal animal, and if required, also aids the use of standard handheld rangefinders which effectively register distances on measuring markers illuminated with spotlights in darkness.

### 2.1 Measuring Flight‐Initiation Distances and Non‐Responses

To demonstrate the capacity of nocturnal FIDs and the taxonomic breadth achievable, we report the mean and standard error of our current successful sampling which includes strictly nocturnal species and those active by day and night. These span both birds and mammals, small (i.e., 15–20 g House Mouse, *Mus musculus*) and large (i.e., 55.2 kg Eastern Grey Kangaroo, *Macropus giganteus*, and 50–70 kg Fallow Deer, *Dama dama*) across temperate Southeastern Australia and tropical Sri Lanka, although additional groups would be equally plausible in other contexts. For many of these species, we believe this to be the first reporting of their FID, and for all nocturnal FIDs, we believe these to be the first reported in darkness. We supplement our observations with a dataset on Silver Gulls (*Chroicocephalus novaehollandiae*), which were sampled slightly differently, by using very low ambient light levels and strong contrast (white feather on green vegetation) by night without the aid of a thermal scope.

Not all individuals responded when approached. This is regularly observed for birds perched in trees or on other structures that are sufficiently high that it mitigates the need to flee, and in our experience, some arboreal mammals in trees showed no response (e.g., Common Ringtail Possum, *Pseudocheirus peregrinus*). We predicted that the presence of ‘non‐responses’ could differ between day and night due to different diel patterns, and/or the potential influence of the cover of darkness of escape responses. We therefore report the proportion of approaches that resulted in ‘non‐responses’ for species sampled both by day and night. These non‐responses are not only an opportunity to investigate behavioural responses and factors which mediate them but could also represent an important sampling consideration.

### 2.2 Starting Distance

Starting distance is a fundamentally important component of any daytime FID measurement (Blumstein 2003). It is almost universally important across taxonomic groups (with some exceptions Cooper 2005; Cooper 2007; Mikula et al. 2019) with longer FIDs being associated with longer StDs. No information exists as to whether this same relationship occurs in darkness, or whether this relationship is the same by day and night. We therefore ran Generalised Linear Models of FID in relation to StD for each species sampled nocturnally. We include the interaction between StD and day versus nighttime sampling for those species sampled during both periods (and dawn or dusk period was defined as daytime). We predicted that FID would increase with StD as is known for daytime responses. Models were initially run with a Poisson distribution, but these data were overdispersed so negative binomial distributions were used. Models were validated through visual inspection of residual values in relation to fitted values and fitted values in relation to each variable in the model.

Ideally, StD would be similar or the same by day and night, to ensure comparability in focal individuals selected for sampling, the distance and time available for decision‐making on the part of the focal animal, and to simplify statistical analysis. Thus, we also compared StD between daytime and nighttime approaches for each species sampled by day and night for which a minimum *n* ≥ 50 for either day or night. We do make this comparison for Silver Gulls noting the above‐mentioned methodological differences.

### 2.3 Sampling Effort Considerations

For any method to be effective in studying an ecological principle, it needs to be able to show variations between contexts, but also estimates should stabilise with increasing sample sizes. Guay et al. (2016) analysed a large dataset of bird FIDs and suggested that 20 replicates per species within a particular context was sufficient for estimated mean FID to stabilise. However, such analyses are limited in the literature, and sampling requirements may meaningfully vary by day and night. We therefore conducted bootstrapping on our dataset for (1) strictly nocturnal species, and (2) for species sampled by both day and night, to consider required sample sizes. Species with > 50 FIDs were used, with 100 subsets sampled of 2–100 FIDs (or the largest number available for the species). The mean and 95% confidence intervals were estimated for each sample size from these 100 repeats. We reported sample size requirements when there was a clear visual stabilisation in FID estimates with decreased confidence intervals.

## 3 Results

**FIGURE 3:**
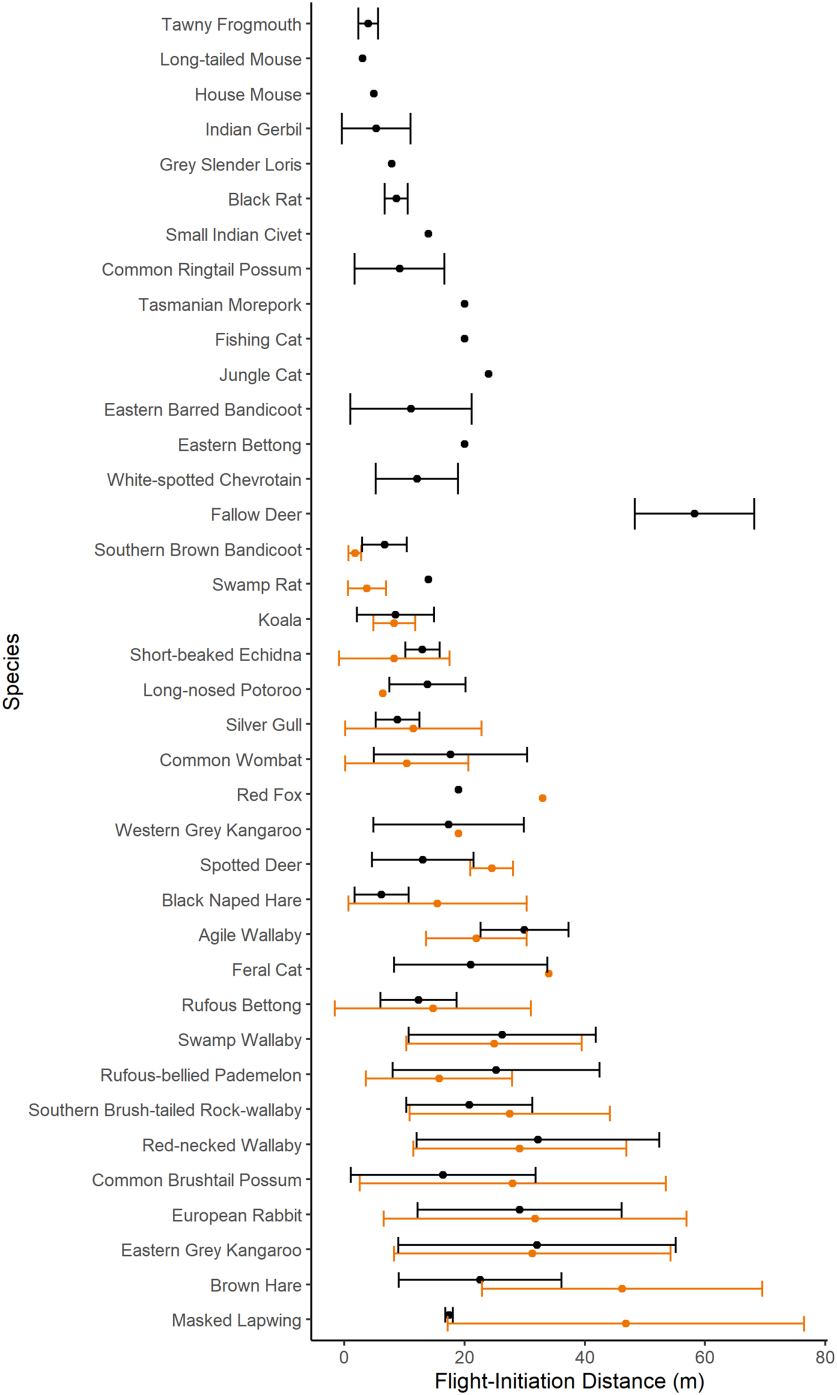
Species mean estimate (±1.96 SE) for flight‐initiation distance for species sampled by day (orange) and night (black). Raw means are presented, uncorrected for Starting Distance (data are provided in Table S1). Strictly nocturnal species are at the top.

We conducted 1469 nighttime FIDs of 34 mammal species and 20 of four bird species (Table S1). These species span large charismatic species (e.g., Eastern Grey Kangaroo, Koala *Phascolarctos cinereus*) to medium‐sized cryptic species (White‐spotted Chevrotain *Moschiola meminna*, Feral Cat *Felis catus*, Fishing Cat *Prionailurus viverrinus*, Jungle Cat *F. chaus*), to small mammals (Black Rat *Rattus rattus*, Swamp Rat *R. lutreolus*, Long‐tailed Mouse *Pseudomys higginsi*) demonstrating the capacity for the technique to be used on a wide variety of species, across many contexts (Figure 3). Silver gulls were also able to be sampled by night without the aid of a thermal scope to enable a further comparison and showed comparable trends. Although this was only possible due to the high contrast between the animals' plumage and background, future work would meaningfully use thermal scopes.

Four species had sufficient data to compare whether FID varied by daytime and nighttime. We found differences for European Rabbits, Eastern Grey Kangaroo and Swamp Wallaby, but not for southern Brush‐tailed Rock Wallaby. Rabbits and kangaroos both had longer FIDs by day when compared to at night (rabbits, daytime *μ* = 29.96 m [95% CI: 27.94–32.14], nighttime = 26.84 [25.03–29.08]; kangaroo, daytime = 33.12 [31.50–34.81], nighttime = 28.79 [25.03–33.12]). In contrast, Swamp Wallaby had longer FIDs by night (nighttime = 28.50 m [24.78–32.79], daytime = 23.34 [21.54–25.28]).

### 3.1 Species Responses to Investigator Approaches

As is the case by day, most animals we approached by night exhibited escape responses; of the 38 species sampled, only seven (18%) exhibited non‐responses (14.8% of all approached by night, Figure S1). Non‐responses occurred both by day and night and the relative proportions were species‐specific (Figure S2). Non‐responders were arboreal and in trees when approached or tame. As is the case by day, there was high variation between species‐specific FIDs and high variation of FIDs within species (Figure 3). We observed similarities in daytime and nighttime FIDs for some species (Eastern Grey Kangaroo, European Rabbit *Oryctolagus cuniculus*, Red‐necked Wallaby *Notamacropus rufogriseus*), yet also observed distinct differences for others (Spotted Deer *Axis axis*, Brown Hare *Lepus europaeus*, Masked Lapwing *Vanellus miles*) (Figure 3).

### 3.2 Starting Distance

**FIGURE 4:**
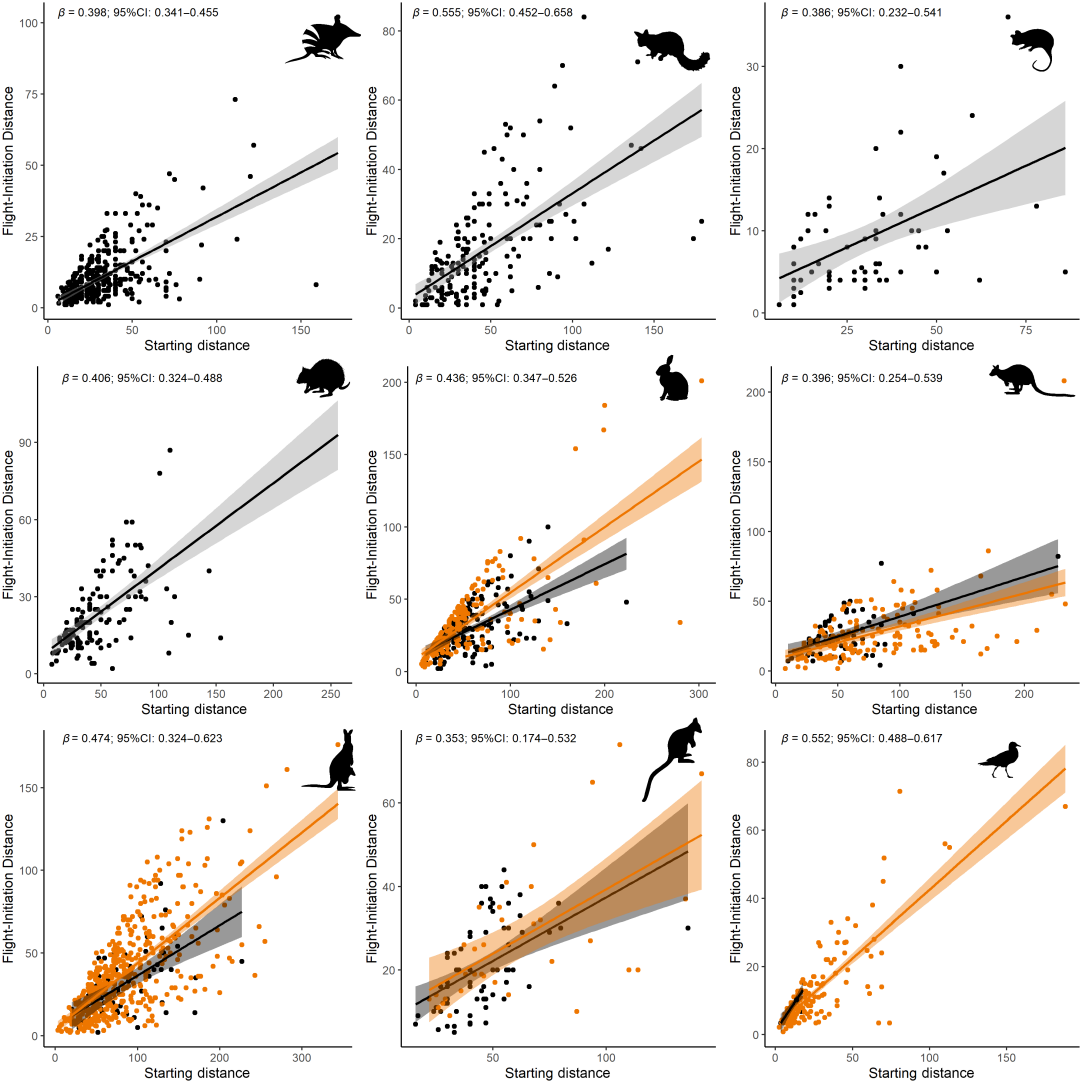
Relationship between starting distance and flight‐initiation distance by day (orange) and night (black). Species include Eastern Barred Bandicoot (top left), Common Brushtail Possum (top middle), Common Ringtail Possum (top right), Rufous‐bellied Pademelon (middle left), European Rabbit (middle middle), Swamp Wallaby (middle right), Eastern Grey Kangaroo (bottom left), Southern Brush‐tailed Rock‐wallaby (bottom middle) and Silver Gull (bottom right).

Starting Distance had a consistent effect on FID with longer StD associated with longer FID for all species including strictly nocturnal species and those active by day and night (Figure 4). For the four species active by day and night, we found no support for an interaction between Starting Distance and day versus night supporting the suggestion that the effect of Starting Distance is consistent across both periods (Figure 4).

### 3.3 Sample Size

**FIGURE 5:**
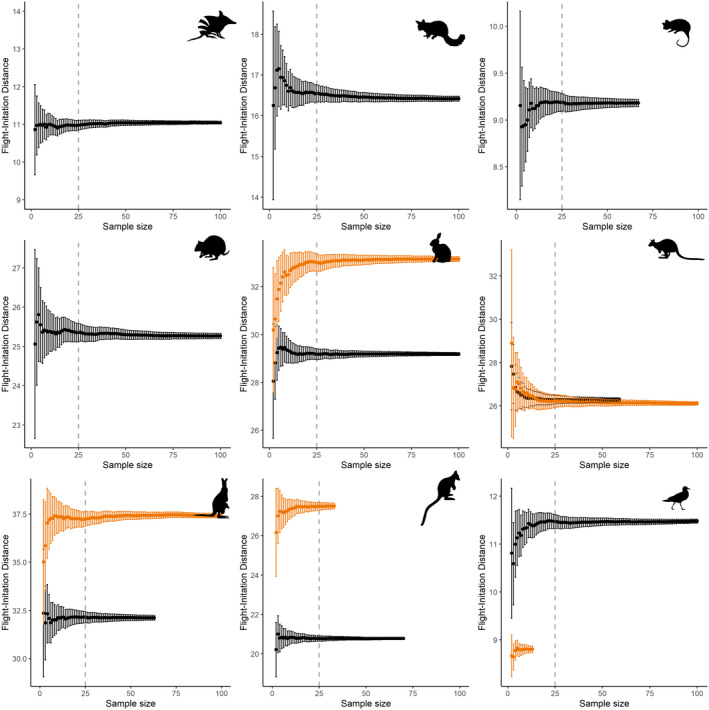
Bootstrapped estimates of flight‐initiation distance and 95% confidence intervals for Eastern Barred Bandicoot (top left), Common Brushtail Possum (top middle), Common Ringtail Possum (top right), Rufous‐bellied Pademelon (middle left), European Rabbit (middle middle), Swamp Wallaby (middle right), Eastern Grey Kangaroo (bottom left), Southern Brush‐tailed Rock‐wallaby (bottom middle) and Silver Gull (bottom right).

We found that, for most species, a sample size of 25 was sufficient for FID estimates to stabilise. For species that were active by day and night, we observed similar sample size requirements (25 samples) irrespective of when sampling occurred (Figure 5). In the absence of data to estimate sampling requirements, we therefore recommend collecting at least 25–50 FIDs per species, per context (or treatment) for comparisons. However, for some species, additional sampling may be required, and researchers should validate their sampling is adequate within their study.

## 4 Discussion

We describe and demonstrate the capacity to conduct efficient, standardised, un‐biased collection of FIDs by night, thereby opening substantial new avenues of investigation across a broad range of taxonomic groups. Our sampling spans large easily identifiable species, alongside smaller and cryptic species, showing the potential applicability of the method across a broad range of taxa inhabiting diverse habitats. In doing so, we present FIDs for species hitherto unsampled, and nighttime FIDs for species for which daytime FIDs only have been available. We also present differences and between‐species variation in day versus nighttime FIDs between taxa—for some taxa FIDs may be the same or similar, for others they differ. Therefore, simple extrapolation of daytime FIDs (readily available) to nighttime FIDs (largely unknown) appears unwise. Factors which mediate these differences or similarities (e.g., sensory modalities, diel predator regimes, different selection pressures), would be the fruitful subjects of future comparative analyses.

The Starting Distances we achieved by night were similar to those by day, suggesting broad comparability. The positive association between FID and StD reported broadly for many taxa by day (Blumstein 2003) also appears to hold for species at night, at least for the taxa we examined. Explanations of the effect of StD on FID vary, but prominent among these is that there is a cost to an animal to remain in a patch under increasing risk (Blumstein 2003). The existence of these effects also implies that animals can judge the distance of an approaching threat by night (but see Dumont et al. 2012). Regardless of the cause, measuring StD by night would seem important for analysis and comparability to both daytime and other nighttime samples. We show it is practical to measure StD by night.

Sample size requirements were similar by day and night for the taxa we modelled and similar to birds by day (Guay et al. 2016). It is critical to consider this in the context of the study system and species because responses could conceivably vary substantially due to many factors (e.g., environmental conditions, vegetation type, level of arboreality, slope). Our recommendation should therefore be taken as a minimum sample size requirement. That said, for most taxa, obtaining a sample size of 25–50 is practical, even by night. While double the human resource is required to sample by night, and additional equipment is required, neither are prohibitive for funded research.

Nocturnal FIDs introduce added complexities when considered against daytime FIDs. For example, in many countries predators of humans are active by night, human visual capacity is severely diminished, and species identification can be more challenging. We propose a range of solutions, yet we acknowledge these are not perfect. For example, communication via two‐way radios to ensure direct approaches is viable; however, species with acute hearing capacity may be able to detect these subtle noises, influencing responses. Hence, single‐observer approaches are desirable, but these are logistically limited to landscape contexts where approaches can be conducted safely (i.e., pastoral landscapes). Approaches that mitigate sound for double‐observer nocturnal approaches, such as communicating via ‘clicking’ the radio receiver to adjust the investigators' direction, rather than talking, could meaningfully be developed to mitigate these limitations.

Nocturnal FIDs offer a diverse range of research possibilities and applications (Gaston 2019; Figure S3). Taxonomically, the substantial diversity of nocturnal species may now be sampled, and diurnal and day/night species may be sampled at night, enabling broader comparative analyses including those which explore differences in these diel ecologies (Gaston 2019). Experimental tests of theoretically derived questions can exploit FIDs in darkness, a key period during which predators and prey may interact and have done so in evolutionary time. Key threatening processes to biodiversity which influence escape in wildlife can be temporally partitioned (e.g., artificial light at night, introduced nocturnal predators) requiring assessments of impacts and adaptations which are now possible. Finally, the increase in nocturnal ecotourism centred around wildlife requires scientifically informed guidelines to minimise impacts on nocturnally active iconic wildlife (Wolf and Croft 2012). This may be especially important given night offers many species of wildlife some respite from human disturbance (Gaynor et al. 2018).

## Author Contributions

**Anthony R. Rendall:** conceptualization (equal), data curation (equal), formal analysis (equal), funding acquisition (equal), investigation (equal), methodology (equal), project administration (equal), resources (equal), software (equal), supervision (equal), validation (equal), visualization (equal), writing – original draft (equal), writing – review and editing (equal). **Roan D. Plotz:** conceptualization (equal), data curation (equal), investigation (equal), methodology (equal), project administration (equal), resources (equal), supervision (equal), validation (equal), visualization (equal), writing – review and editing (equal). **Kaori Yokochi:** conceptualization (equal), data curation (equal), funding acquisition (equal), investigation (equal), methodology (equal), project administration (equal), resources (equal), software (equal), supervision (equal), validation (equal), visualization (equal), writing – review and editing (equal). **Joel Krauss:** data curation (supporting), investigation (supporting), project administration (supporting), writing – review and editing (supporting). **Aaron Pengelly:** data curation (supporting), investigation (supporting), project administration (supporting), writing – review and editing (supporting). **Sam A. Di Stefano:** data curation (supporting), investigation (supporting), project administration (supporting), writing – review and editing (supporting). **Sarah Swindell:** data curation (supporting), project administration (supporting), writing – review and editing (supporting). **Kithsiri Ranawana:** data curation (equal), project administration (equal), writing – review and editing (equal). **Dulan R. Vidanapathirana:** data curation (equal), project administration (equal), writing – review and editing (equal). **Michael A. Weston:** conceptualization (equal), data curation (equal), funding acquisition (equal), investigation (equal), methodology (equal), project administration (equal), resources (equal), supervision (equal), validation (equal), visualization (equal), writing – original draft (equal), writing – review and editing (equal).

## Ethics Statement

Animal ethics approvals were obtained (Deakin University Animal Ethics Committee approvals B32‐2012, B11‐2015, B10‐2018, B08‐2021, B32‐2022), and fieldwork was conducted under permits in Australia (10008731, 10010123, 10010713, SL101622, AA‐0001059, TFA22300, TFA 23046, Y26590‐1) and Sri Lanka (WL/3/2/61/15; WL/3/2/43/2023; NFSRC/2023/02/87/P6).

## Conflicts of Interest

The authors declare no conflicts of interest.

## Supporting information


Appendix S1.

## Data Availability

All data used to conduct the analyses, including online zip files which reflect the cited online repositories within the manuscript illustrating how nocturnal flight‐initiation distances were effectively conducted using thermal technology on a variety of animal species, are available at Zenodo Research Repository: DOI: https://doi.org/10.5281/zenodo.11081716 and https://zenodo.org/records/11081835.
