# Supplementary material for: Lifting the Veil of Darkness: Thermal Technology Facilitates Collection of Flight‐Initiation Distances by Night
Source: Ecol Evol. 2024 Nov 19;14(11):e70450. doi: 10.1002/ece3.70450 (PMC11576410; doi:10.1002/ece3.70450)
Supplement: Supplementary file 1 — Appendix S1. [file ECE3-14-e70450-s001.docx]

**SUPPLEMENTRAY MATERIAL**

**Lifting the veil of darkness: thermal technology facilitates collection of flight-initiation distances by night**

**Table S1.** Samples sizes and raw means and standard errors for Flight-initiation Distances by day and night. *indicated arboreal species. § only non-breeding free flying individuals sampled. Blanks indicate no data available.

| **Common name** | **Scientific name** | **Number of FIDs**  **Night (Day)** | **Nocturnal**  **µ (SE)** | **Diurnal**  **µ (SE)** |
| --- | --- | --- | --- | --- |
| Eastern Barred Bandicoot | *Perameles gunnii* | 372 | 11.07 (0.52) |  |
| Common Brushtail Possum* | *Trichosurus vulpecula* | 260 | 16.41 (1.14) |  |
| European Rabbit | *Oryctolagus cuniculus* | 165 (140) | 29.17 (1.33) | 31.70 (2.13) |
| Common Ringtail Possum* | *Pseudocheirus peregrinus* | 154 | 9.17 (0.92) |  |
| Rufous-bellied Pademelon | *Thylogale billardierii* | 121 | 25.24 (1.57) |  |
| Southern Brush-tailed Rock-wallaby | *Petrogale penicillata* | 70 (33) | 20.78 (1.25) | 27.50 (2.90) |
| Eastern Grey Kangaroo | *Macropus giganteus* | 63 (377) | 32.06 (2.91) | 31.20 (1.18) |
| Swamp Wallaby | *Wallabia bicolor* | 60 (153) | 26.26 (2.02) | 24.90 (1.18) |
| Red-necked Wallaby | *Notamacropus rufogriseus* | 48 (24) | 32.21 (2.92) | 29.20 (3.61) |
| Common Wombat | *Vombatus ursinus* | 39 (20) | 17.66 (2.07) | 10.40 (2.28) |
| Agile Wallaby | *Notamacropus agilis* | 21 (22) | 29.95 (1.60) | 22.00 (1.78) |
| Rufous Bettong | *Aepyprymnus rufescens* | 17 (7) | 12.35 (1.54) | 14.70 (6.17) |
| Long-nosed Potoroo | *Potorous tridactylus* | 15 (1) | 13.79 (1.64) | 6.40 (NA) |
| Koala* | *Phascolarctos cinereus* | 11 (8) | 8.48 (2.42) | 8.30 (1.22) |
| Brown Hare | *Lepus europaeus* | 9 (4) | 22.56 (4.51) | 46.20 (11.6) |
| Indian Gerbel | *Tatera indica* | 6 | 5.29 (2.32) |  |
| Southern Brown Bandicoot | *Isoodon obesulus* | 6 (2) | 6.67 (1.52) | 1.75 (0.75) |
| Fallow Deer | *Dama dama* | 4 | 58.25 (4.97) |  |
| Spotted Deer | *Axis axis* | 4 (2) | 13.01 (4.22) | 24.50 (2.5) |
| Western Grey Kangaroo | *Macropus fuliginosus* | 3 (1) | 17.33 (7.22) | 19.00 (NA) |
| White-spotted Chevrotain | *Moschiola meminna* | 3 | 12.06 (3.97) |  |
| Black Rat | *Rattus rattus* | 2 | 8.64 (1.36) |  |
| Black-naped Hare | *Lepus nigricollis* | 2 (2) | 6.18 (3.18) | 15.50 (10.5) |
| Feral Cat | *Felis catus* | 2 (1) | 21.00 (9.00) | 34.00 (NA) |
| Grey Slender Loris* | *Loris lydekkerianus* | 1 | 7.87 (NA) |  |
| Short-beaked Echidna | *Tachyglossus aculeatus* | 2 (21) | 13.00 (2.00) | 8.30 (2.0) |
| Eastern Bettong | *Bettongia gaimardi* | 1 | 20.00 (NA) |  |
| Fishing Cat | *Prionailurus viverrinus* | 1 | 20.00 (NA) |  |
| House Mouse | *Mus musculus* | 1 | 4.92 (NA) |  |
| Jungle Cat | *Felis chaus* | 1 | 24.00 (NA) |  |
| Long-tailed Mouse | *Pseudomys higginsi* | 1 | 3.00 (NA) |  |
| Red Fox | *Vulpes vulpes* | 1 (1) | 19.00 (NA) | 33.00 (NA) |
| Small Indian Civet | *Viverricula indica* | 1 | 14.00 (NA) |  |
| Swamp Rat | *Rattus lutreolus* | 1 (2) | 14.00 (NA) | 3.75 (2.25) |
| Masked Lapwing§ | *Vanellus miles* | 2 (163) | 17.40 (0.43) | 46.80 (2.32) |
| Silver Gull§ | *Chroicocephalus novaehollandiae* | 13 (178) | 8.83 (1.01) | 11.50 (0.85) |
| Tawny Frogmouth*§ | *Podargus strigoides* | 4 | 3.93 (0.82) |  |
| Tasmanian Morepork*§ | *Ninox novaeseelandiae* | 1 | 20.00 (NA) |  |


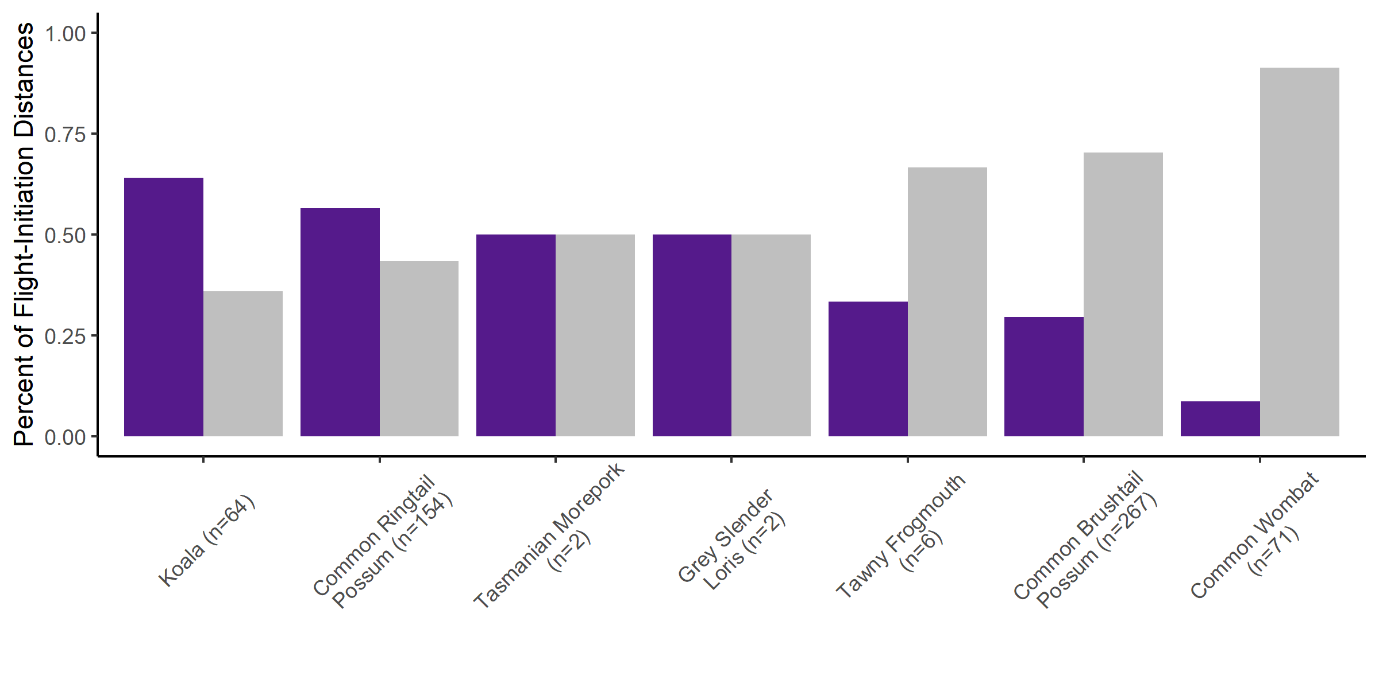


**Fig. S1.** The proportion of Flight-Initiation Distances that had no escape response (purple) compared to those that showed an escape response (grey).


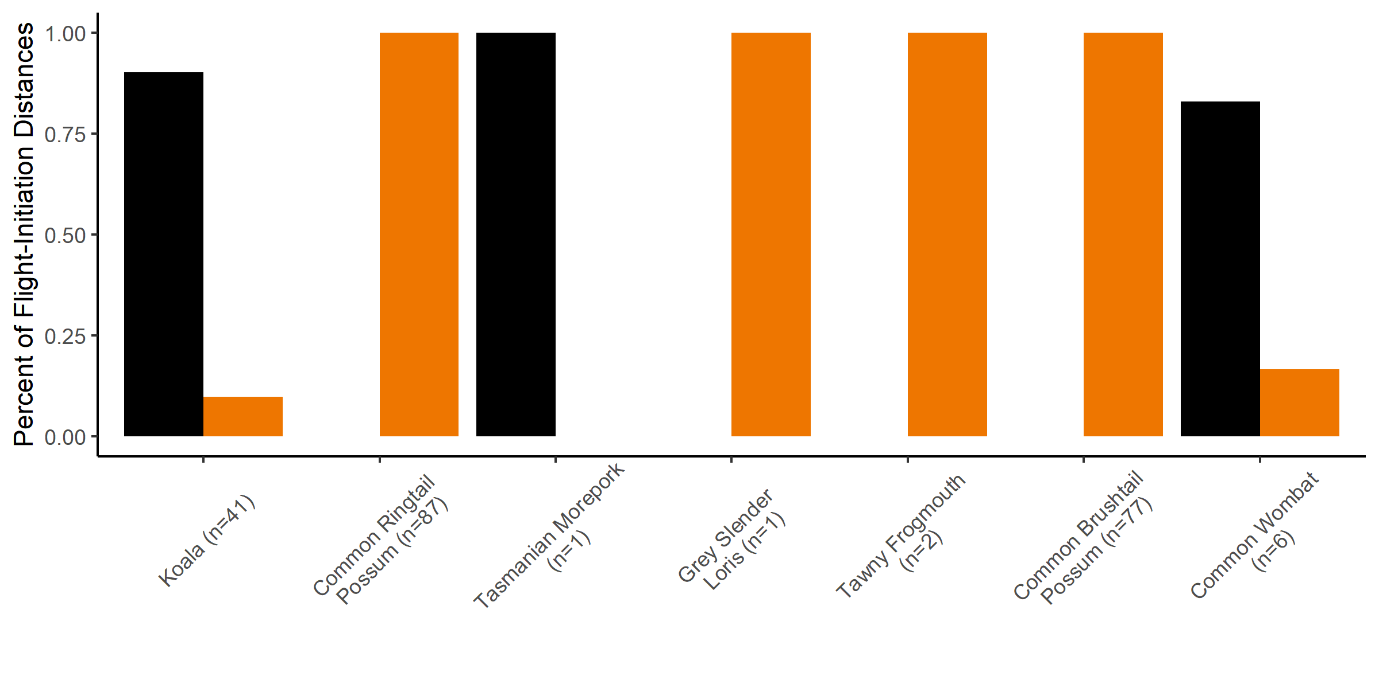


**Fig. S2.** The proportion of approaches that resulted in an escape response at nighttime (black) and during the daytime (orange).

**Fig. S3.** Possible applications of nighttime Flight-Initiation Distances that offer substantial realms for future research.
